# General Practice Registrars’ Management of and Specialist Referral Patterns for Atopic Dermatitis

**DOI:** 10.5826/dpc.1101a118

**Published:** 2021-01-29

**Authors:** Anneliese Willems, Amanda Tapley, Alison Fielding, Vivian Tng, Elizabeth G. Holliday, Mieke L. van Driel, Jean I. Ball, Andrew R. Davey, Kristen FitzGerald, Neil A. Spike, Parker J. Magin

**Affiliations:** 1 Eastern Victoria GP Training, General Practice Training Organisation, Melbourne, VIC, Australia; 2 The University of Melbourne, Department of General Practice, Melbourne, VIC, Australia; 3 The University of Newcastle, School of Public Health and Medicine, Callaghan, NSW, Australia; 4 GP Synergy, Regional Training Organisation, NSW & ACT Research and Evaluation Unit, Newcastle, NSW, Australia; 5 Department of Dermatology, John Hunter Hospital, Newcastle, NSW, Australia; 6 The University of Queensland Faculty of Medicine, Primary Care Clinical Unit, Brisbane, QLD, Australia; 7 Hunter Medical Research Institute, Clinical Research Design, IT and Statistical Support Unit (CReDITSS), New Lambton, NSW, Australia; 8 University of Tasmania, School of Medicine, Hobart, TAS, Australia; 9 General Practice Training Tasmania (GPTT), Regional Training Organisation, Hobart, TAS, Australia

**Keywords:** atopic dermatitis, eczema, referral and consultation, general practice, dermatologist

## Abstract

**Background:**

Atopic dermatitis (AD) is a common presentation in the general practice (GP) setting. Implementation of appropriate referral pathways is instrumental for best patient care and is an essential skill for Australian GP registrars.

**Objectives:**

We aimed to explore the prevalence and associations of GP registrar referrals to specialists for AD management.

**Methods:**

A cross-sectional analysis utilizing data from the Registrar Clinical Encounters in Training (ReCEnT) project, an ongoing cohort study that documents in-consultation clinical and educational experience of Australian GP registrars. Registrar, patient, and consultation factors associated with referrals for AD were established using logistic regression.

**Results:**

A total of 2,783 registrars (96% response rate) provided data from 381,180 consultations from 2010 to 2019. A total of 3,285 (0.55%) of 595,412 diagnoses managed were AD, of which 222 (6.8%) resulted in referral. Of these referrals, 70% were to dermatologists, 17% to allergists/immunologists, and 10% to pediatricians. Associations of referral included registrar female gender, patient age, longer consultation duration; an established (rather than new) AD diagnosis; supervisor advice being sought; and learning goals being generated.

**Conclusions:**

Both registrar and patient factors influence AD referral patterns. Registrars referred established rather than newly diagnosed AD, suggesting a level of comfort in initial management. Referral was associated with longer consultations, seeking supervisor advice, and generation of learning goals—suggesting these are more complex presentations and, possibly, registrar learning opportunities. A significant proportion of referrals were to non-dermatologist specialists. The implication of this for optimal patient care is a subject for further study.

## Introduction

Atopic dermatitis (AD) is associated with psychological, social, and quality-of-life effects in patients and their families [1,2]. AD has a 12-month prevalence of 16%–17% in childhood and is characterized by chronic inflammation and pruritus [ 3]. While AD is predominantly a childhood illness [4], an estimated 50% of those affected by AD in their early years go on to have symptoms into their teenage years and adulthood [5].

Given its prevalence, AD represents a common presentation in the primary care setting. The majority of AD is mild and may be managed in primary care, rather than in specialty practice [6]. Skin presentations represent 11% of all problems encountered by GP registrars. Of these, 12% are “dermatitis,” including AD [7].

AD is generally a straightforward clinical diagnosis [8,9], and the treating clinician must first exclude other conditions [6]. Patient education underpins treatment and all cases should be treated with emollients [8]. Topical corticosteroids and, when appropriate, antimicrobial and antiseptic measures, comprise the next level of therapy [8]. This management can be appropriately delivered in primary care. In moderate-severe disease, referral may be necessary for access to additional therapies such as narrowband UVB phototherapy or oral immunosuppressive therapies [10]. Additionally, a specialist’s opinion may occasionally be needed for clarification of diagnosis or for exploration of exacerbating factors, such as allergy [6].

With a breadth of management options available according to severity of AD disease, it is important for clinicians to have confidence in diagnosis and early stages of management [9]. Similarly, clinicians should be competent in recognizing disease severity and have a good understanding of the indications for referral [6]. This may be problematic in primary care. Some studies have suggested that GPs may lack confidence in diagnosis [11,12] and management of skin disease [11,13,14]. GPs and vocational trainees in specialist general practice (GP registrars) have limited formal training in managing skin disease [7,13,15]. There is relatively little teaching of dermatology during medical school [16,17]. Dermatology placements are infrequent in junior doctor rotations and, as such, the bulk of dermatology learning takes place in-practice during postgraduate terms [16]. Consequently, skin disease remains a learning-need for GP registrars, and GP registrars find skin consultations problematic compared to non-skin consultations [7].

There is also a lack of evidence regarding appropriate circumstances for a GP referral of skin diseases [18,19]. Given the historical challenges of GP registrar management of skin disease, the frequency of AD in Australia, and the challenges in providing appropriate referrals, we sought to address an evidence gap. In this study we sought to explore the nature, frequency, and associations of GP registrar specialist referrals for AD.

## Materials and Methods

We performed a cross-sectional analysis of data within the Registrar Clinical Encounters in Training (ReCEnT) project. ReCEnT documents the in-consultation educational and clinical experiences of GP registrars.

### GP Vocational Training

GP training in Australia operates under an apprenticeship-like model wherein registrars are supervised by senior accredited GPs within an accredited practice environment. Training entails at least 3 6-month, full-time-equivalent, community -based general practice terms.

### ReCEnT

Registrars complete 3 cycles of ReCEnT data collection during training, once each term. Details of 60 consecutive consultations are collected on paper-based Case Report Forms. From these consultations, problems managed and referrals made are coded according to the International Classification of Primary Care (second edition) classification system (ICPC-2 Plus) [20]. Registrar and practice variables are also collected every 6 months. ReCEnT was conducted in up to 5 Regional Training Providers (RTP), across 5 states, during 2010–2015, and in 3 Regional Training Organisations (RTO), across 3 states and 1 territory, from 2016, following a major restructuring of Australian GP vocational training.

### This Study

Data from 2010–2019 is included in the current study. For this study, our analyses were confined to consultations coded as “dermatitis, atopic,” “eczema,” and “eczema, infantile.” See Supplementary Table 1 for a complete list of inclusion and exclusion ICPC-2 Plus codes.

#### Outcome Factor

The outcome was “specialist referral made.”

#### Independent Variables

Independent variables included in these models included registrar, practice, patient, consultation and educational factors. Patient factors were: patient age group, patient gender, Aboriginal or Torres Strait Islander status, and non-English speaking background status. Registrar factors were: registrar age and gender, full-time or part-time employment status, the term of GP training, whether the registrars had worked at their current practice previously, and whether they had qualified as a doctor in Australia. Practice factors were: the size of the practice, whether the practice was fully bulk-billing (that is, no fee charged to the patient), the rurality of the practice, the RTP or RTO the registrar was enrolled with (hereafter “region”), and the socio-economic index for areas,-index of relative socioeconomic disadvantage (SEIFA-IRSD) in which the practice was located. Consultation factors were: the duration of consultation, number of problems managed within the consultation, whether AD was a new problem, whether the registrar sought assistance for diagnosis or management of the problems managed, whether pathology was ordered, whether follow-up was ordered, whether learning goals were generated, and which medications, if any, were prescribed.

#### Statistical Analyses

Statistical analysis was at the level of problem/diagnosis. The proportion of problems/diagnoses that were atopic dermatitis and, of those, the proportion that were referred were calculated with 95% confidence intervals (CI) and were adjusted for repeated measures within registrars.

Descriptive statistics included frequencies for categorical variables and mean with standard deviation for continuous variables. The frequencies of categorical variables were compared between outcome categories using chi-square tests for all variables, except when Fisher’s exact test was used (due to an expected count less than 5 in 25% or more cells). For continuous variables, means were compared using a t test.

Logistic regression has been used within the generalized estimating equations (GEE) framework to account for repeated measures within registrars. An exchangeable working correlation structure was assumed. Univariate analyses were conducted on each covariate with the outcome. Covariates with a univariate P value < 0.20 were considered for inclusion in the multiple regression model.

Once the model with all significant covariates was fitted, model reduction was assessed. Covariates that were no longer significant (at P < 0.2) in the multivariable model were tested for removal from the model. If the covariate’s removal did not substantively change the resulting model, the covariate was removed from the final model. A substantive change to the model was defined as any covariate in the model having a change in the effect size (odds ratio) of greater than 10%.

Diagnostic tests were conducted to assess goodness of fit, using the Hosmer-Lemeshow test for logistic models. Predictors were considered statistically significant if the P value was < 0.05.

Statistical analyses used STATA 14.1 (StataCorp, College Station, TX, USA) and SAS V9.4 (SAS Institute Inc., Cary, NC, USA).

This project has ethics approval through an appropriate Human Research Ethics Committee.

## Results

In total, 2,783 registrars (96.1% response rate) provided data from 381,180 consultations from 2010–2019, including 595,412 problems managed. Table 1 shows the demographics of participating registrars.

Of all problems, 3,285 (0.55% [95% CI: 0.53, 0.57]) were AD. Of all AD problems, 222 (6.8% [95% CI: 6.0, 7.7]) were referred. Of these referrals, 70% were to dermatologists (Table 2), 17% to allergists/immunologists, 10% to pediatricians, and 3% to a clinic without specifying the specialist.

The characteristics associated with specialist referral are presented in Table 3. As well as the associations with referral, overall findings of note are that for 9.7% of AD problems, supervisor advice or assistance was sought and for 18% of AD problems learning goals were generated.

The results of univariate and multivariable logistic regression are presented in Table 4.

Statistically significant multivariable associations of an AD problem being referred to a specialist included registrar female gender (OR 1.49 [95% CI: 1.05, 2.12]) and being Australian-trained (OR 2.02 [1.18, 3.48]); patient age (patients aged 0–1 year less likely to be referred [OR 0.58 [0.34, 0.98] compared to patients aged 2–12 years); AD being an existing problem (OR 0.28 [0.18, 0.43] for a new problem); pathology being ordered (OR 2.48 [1.08, 5.69]); and learning goals being generated (OR 2.57 [1.69 3.90]).

Also significant in multivariable analyses, less non-AD problems, on average, were addressed in consultations resulting in referral (OR 0.57 [95% CI: 0.43, 0.75]), and these consultations were longer (OR 1.05 [95% CI: 1.02, 1.07] for each additional minute of consultation duration). In univariate analysis (Table 3), referral was associated with an estimated average increase of 3 minutes in AD consultation duration.

In AD consultations resulting in referral, supervisors were more likely to provide the registrar with advice or assistance (20% versus 9%), which was significant on multivariable analysis (OR 1.73 [95% CI: 1.06, 1.83]).

## Discussion

The associations of GP and GP registrar referrals for AD have not been well investigated. To our knowledge this is the first analysis exploring Australian GP registrars’ (or GP trainees in other countries) referrals for AD. This study provides several significant findings pertaining to registrar engagement and confidence with AD, frequency of referral, and specialist choice.

### Summary of Main Findings

Registrars referred 6.8% of AD cases. The dermatologist was the preferred specialist to manage AD, with 70% of cases referred to this specialty, but a relatively high percentage of cases (17%) were referred to allergists/immunologists.

Prominent associations of referrals included longer consultations, a preexisting AD diagnosis, and consultations in which fewer issues were managed. There was increased supervisor involvement for consultations in which AD was referred and considerably increased learning-goal generation for these consultations.

### Interpretation of Findings and Comparison With Previous Literature

The context for our finding of referral of 6.8% of AD problems is that GPs have previously been shown to refer 10.3% of all problems managed [21]. In an earlier analysis of this Australian registrar population, we found a referral rate for skin problems of 8.0%. This earlier analysis also found that registrars were 38% (odds ratio [OR] 0.62) less likely to refer skin problems compared with non-skin problems.

Our results, though, suggest AD remains a significant learning-need for GP registrars. Compared to all problems seen (in previous analyses from ReCEnT), when presented with a diagnosis of AD, a GP registrar is more likely to seek supervisor advice or assistance (9.7% compared to 6.9% for all problems [22]), and generate learning-goals (18.4% compared to 16.6% for all problems [23]). This is consistent with previous evidence that diagnosing skin diseases (including AD) is an area that receives less attention throughout undergraduate, postgraduate, and specialist training for GP trainees [7,16,17,24]. Qualified GPs are also reported to have some difficulty with AD management [13].

The relative frequency of referrals of an existing rather than a new AD problem/diagnosis suggests that, while registrars may need considerable training to manage AD, they may nevertheless be comfortable with AD diagnosis and initial management. Despite presenting some challenges for the early-career GP, AD is most often a straightforward diagnosis, is mostly of mild or moderate severity, and can be managed appropriately by the GP using first-line modalities. It is then likely that the advice and assistance of their supervisor may support registrars’ management of AD in the context of them having considerable learning-needs in the area.

It was also apparent that referral for AD may be associated with a more complex or problematic AD presentation: longer consultation duration, greater learning-goal generation, more supervisor assistance sought, more pathology ordered, and fewer non-AD problems addressed in the index consultation.

Another notable finding of this study was that while the dermatologist is the specialist of choice for AD, a relatively large proportion of patients are referred to allergists/immunologists. The roles of each of these specialists as a part of the multidisciplinary team have previously been recognized in the care of moderate-severe AD [25]. Significantly, the choice of specialist has been shown to influence management practices [26, 27]. Allergists and dermatologists diverge in their recommendations for systemic treatments, adjunctive therapies, and preventative measures [25]. Given the natural emphasis of their training, allergists/immunologists are more likely to focus on preventative strategies and potential allergic triggers [27].

Our results suggest registrars are quite frequently prioritizing allergy pathways in AD management. The 17% referred to allergists/immunologists may reflect the rise in primary care recognition of the association of eczema with food allergy. A review of studies by Werfel et al. in 2007 found a prevalence of food allergy in 33%–63% of cases [28]. The exact relationship between food allergy and AD remains controversial. Food allergy as an aggravating factor of AD in infants and young children is well accepted [29], but there is a lack of consensus regarding food allergy as an exacerbating factor versus a cause [25, 30].

It has previously been suggested that primary care providers overemphasize the role of food allergy in AD [30]. Our results also suggest a registrar emphasis on a possible allergic component to AD. Overemphasis on allergies versus the overarching etiology of AD can lead to miscommunication, which results in a parent erroneously believing that a food allergy is causing their child’s AD [30]. This can then lead to restrictive diets, risking possible nutritional deficiencies, and diversion from the basics of optimizing AD management such as education, skin care, and optimal use of topical corticosteroids [30].

Referral rates for AD in this study are reflective of the existing literature, as is our finding of AD as a persistent learning-need for GP registrars. As yet, there is limited literature to compare our findings of registrar AD referral pathways, and the implications of specialist choice on clinical outcomes.

### Strengths and Limitations

This study has a number of strengths, in particular, its high response rate (96.1%) and broad data set (595,412 data points) and geographic distribution throughout areas classified as urban, rural, and remote across multiple Australian states. As such, findings are generalizable across Australia and, potentially, internationally. A limitation of the analysis was the limited clinical context provided within the consultation data. We did not have contextual information on severity of AD, of AD past history, of medicine regimens, of concurrent conditions and, thus, were unable to ascertain if individual referrals were appropriate for care escalation.

### Implications for Practice and Future Research

Our findings suggest that registrars are engaging with diagnosis and management of AD, but that AD (as with skin disease generally), remains a priority learning-need for registrars. Continued reevaluation of postgraduate dermatology curricula would best focus on management components and understanding of appropriate indications for referral. Clarification, such as through referral pathways, for specific specialist involvement would help to guide appropriate specialist referral according to patient presentation. Furthering awareness of and access to multidisciplinary clinics, wherein a variety of specialists are available, may also help to optimize GP registrars’ contribution to AD management.

## Conclusions

Our findings show that GP registrars are engaging with AD, yet it is apparent this is still a learning-need for many registrars. Registrar education could include appropriate referral pathways and further clarification regarding the role of allergy in AD.

## Supplementary Information

**Supplementary Table 1:** 

Included Diagnostic Codes	Excluded Diagnostic Codes
Dermatitis, atopic Eczema Eczema, infantile	Rash; atopic Dermatitis; flexural Dermatitis; allergic Dermatitis

## Figures and Tables

**Table 1:** Demographics of Participating GP Registrars and Their Practices

Registrar Variables (n=2783)		n (%)
Registrar gender	Male	1,055 (37.9)
	Female	1,728 (62.1)
Qualified as doctor in Australia	Yes	547 (19.8)
	No	2,223 (80.3)
Pathway registrar enrolled in	General	1,930 (70.0)
	Rural	826 (30.0)
**Registrar Round/Practice Variables (n=6414)**		
Registrar age (years)	Mean ± SD	32.6 (6.3)
Registrar works Full-time or Part-time	Full-time	4,770 (77.1)
	Part-time	1,420 (22.9)
Registrar training term	Term 1	2,640 (41.2)
	Term 2	2,091 (32.6)
	Term 3	1,683 (26.2)
Practice rurality	Major city	3,983 (62.7)
	Inner regional	1,633 (25.7)
	Outer regional remote	732 (11.5)
Practice SEIFA-IRSD	Mean ± SD	5.5 (2.8)
Practice routinely bulk bills	Yes	1,784 (28.1)
	No	4,566 (71.9)
Registrar worked at practice previously	Yes	1,343 (21.2)
	No	4,988 (78.8)
Practice size	Small (1–5 GPs)	2,371 (38.4)
	Large (6–10+ GPs)	3,811 (61.6)

GP = general practice; SD = standard deviation; SEIFA-IRSD = socio-economic index for area – index of relative socioeconomic disadvantage.

**Table 2:** Referrals for Atopic Dermatitis (n=222)

Specialist	Frequency	Percentage
Dermatologist referral	153	70
Allergist/immunologist referral	38	17
Pediatrician referral	21	9.6
Referral to clinic/center	4	3.2
Other	3	1.4
**Total**	**219**	**100**

Note: Three referrals were excluded, as they did not specify the referral type and hence have been removed from analysis.

**Table 3:** Characteristics by Referral Status in Atopic Dermatitis Consultations (n = 381,180)

			Referral for Atopic Dermatitis		
Factor Group	Variable	Class	No	Yes	P value
Patient factors	Patient age group	0–1 years	655 (22%)	32 (15%)	0.12
		2–12 years	834 (28%)	67 (31%)	
		13–24 years	542 (18%)	49 (23%)	
		25–44 years	541 (18%)	38 (18%)	
		45+ years	441 (15%)	30 (14%)	
	Patient gender	Male	1,318 (44%)	93 (43%)	0.89
		Female	1,677 (56%)	121 (57%)	
	NESB	No	2,589 (90%)	187 (88%)	0.40
		Yes	290 (10%)	25 (12%)	
	Patient/practice status	Existing patient	1,009 (34%)	88 (40%)	0.12
		New to registrar	1,722 (57%)	111 (51%)	
		New to practice	272 (9%)	20 (9%)	
	Aboriginal or Torres Strait Islander	No	2,826 (99%)	210 (99.5%)	0.52
		Yes	38 (1%)	1 (0.5%)	
Registrar factors	Registrar gender	Male	1,131 (37%)	69 (31%)	0.078
		Female	1,932 (63%)	153 (69%)	
	Registrar full-time or part-time	Part-time	733 (25%)	57 (27%)	0.48
		Full-time	2,249 (75%)	156 (73%)	
	Term	Term 1	1,194 (39%)	94 (42%)	0.39
		Term 2	1,122 (37%)	71 (32%)	
		Term 3	747 (24%)	57 (26%)	
	Worked at practice previously	No	2,383 (79%)	180 (82%)	0.33
		Yes	631 (21%)	40 (18%)	
	Qualified as doctor in Australia	No	503 (16%)	29 (13%)	0.18
		Yes	2,547 (84%)	192 (87%)	
	Registrar age	Mean (SD)	32 (6)	32 (6)	0.74
Practice factors	Practice size	Small	1,069 (36%)	76 (36%)	0.90
		Large	1,906 (64%)	138 (64%)	
	Practice routinely bulk bills	No	2,132 (71%)	151 (69%)	0.49
		Yes	879 (29%)	69 (31%)	
	Rurality	Major city	2,075 (69%)	164 (76%)	0.090
		Inner regional	685 (23%)	41 (19%)	
		Outer regional remote	262 (9%)	12 (6%)	
	Region	Region 1	518 (17%)	28 (13%)	0.37
		Region 2	139 (5%)	14 (6%)	
		Region 3	374 (12%)	22 (10%)	
		Region 4	1,300 (42%)	97 (44%)	
		Region 5	28 (0.9%)	3 (1%)	
		Region 6	506 (17%)	44 (20%)	
		Region 7	198 (6%)	14 (6%)	
	SEIFA-IRSD	Mean (SD)	6 (3)	6 (3)	0.098
Consultation factors	New problem seen	No	1,791 (65%)	166 (85%)	<0.001
		Yes	959 (35%)	30 (15%)	
	Sought help any source	None	2,367 (77%)	138 (62%)	<0.001
		Supervisor	274 (9%)	45 (20%)	
		Other sources	422 (14%)	39 (18%)	
	Pathology ordered	No	3,004 (98%)	211 (95%)	0.004
		Yes	59 (2%)	11 (5%)	
	Follow-up ordered	No	1,963 (64%)	131 (59%)	0.13
		Yes	1,100 (36%)	91 (41%)	
	Learning goals generated	No	2,339 (82%)	128 (61%)	<0.001
		Yes	523 (18%)	81 (39%)	
	Consultation duration	Mean (SD)	17 (8)	20 (9)	<0.001
	Number of problems	Mean (SD)	2 (1)	2 (1)	<0.001

NESB = Non-English speaking background; SEIFA-IRSD = socio-economic index for areas – index of relative socioeconomic disadvantage; SD = standard deviation.

**Table 4:** Characteristics Associated With a Referral Being Made for Atopic Dermatitis

			Univariate		Adjusted	
Factor Group	Variable	Class	OR [95% CI]	P value	OR [95% CI]	P Value
Patient factors	Patient age group Comparator 2–12 years	0–1 years	0.61 (0.39, 0.95)	0.027	0.58 (0.34, 0.98)	0.0417
		13–24 years	1.13 (0.76, 1.67)	0.55	1.02 (0.63, 1.63)	0.9444
		25–44 years	0.87 (0.58, 1.32)	0.52	0.91 (0.55, 1.50)	0.6988
		45+ years	0.85 (0.54, 1.32)	0.47	1.23 (0.73, 2.05)	0.4358
Registrar factors	Registrar gender	Female	1.30 (0.97, 1.74)	0.078	1.49 (1.05, 2.12)	0.0269
	Qualified as doctor in Australia	Yes	1.31 (0.88, 1.93)	0.18	2.02 (1.18, 3.48)	0.0110
Practice factors	SEIFA-IRSD		1.04 (0.99, 1.10)	0.098	1.03 (0.98, 1.10)	0.2550
Consultation factors	New problem seen	Yes	0.34 (0.23, 0.50)	<.001	0.28 (0.18, 0.43)	<.001
	Sought help any source	Other sources	1.57 (1.09, 2.27)	0.016	1.19 (0.73, 1.97)	0.48
	Comparator: None	Supervisor	2.82 (1.97, 4.03)	<.001	1.73 (1.06, 2.83)	0.030
	Pathology ordered	Yes	2.65 (1.36, 5.17)	0.004	2.48 (1.08, 5.69)	0.031
	Follow-up ordered	Yes	1.24 (0.94, 1.64)	0.13	0.75 (0.52, 1.09)	0.13
	Learning goals generated	Yes	2.83 (2.10, 3.82)	<.001	2.57 (1.69, 3.90)	<.001
	Consultation duration		1.03 (1.02, 1.05)	<.001	1.05 (1.02, 1.07)	<.001
	Number of problems		0.69 (0.58, 0.83)	<.001	0.57 (0.43, 0.75)	<.001

SEIFA-IRSD = socio-economic index for areas – index of relative socioeconomic disadvantage
